# Computer-mediated Focus Groups to Advance Aging Research: Challenges and Opportunities.

**DOI:** 10.1093/geroni/igab046.3061

**Published:** 2021-12-17

**Authors:** David Coon, Abigail Gomez-Morales, phil Carll, Lourdes Cordova, allison Glinka, Socorro Gonzalez-Piles

**Affiliations:** Arizona State University, Phoenix, Arizona, United States

## Abstract

When compared to in-person offerings, fewer focus groups to date have been conducted with user-friendly technologies to help reach diverse communities of older adults with chronic health conditions (e.g., Alzheimer’s disease, Type II diabetes, Parkinson’s) and the family caregivers and professional providers who assist them. The current project describes the adaptations needed to deliver successful computer-mediated focus groups via videoconference, thereby providing solutions to barriers faced by participants who often cannot attend in-person because they are housebound due to transportation or financial barriers, live in rural areas or reside too far from focus group offerings, or work full or part-time and face scheduling conflicts. During the pandemic, we successfully recruited diverse groups of family caregivers, care recipients, and professional providers into computer-mediated focus groups. Caregivers (83%) and care recipients (17%) between 34 to 90 years old (N=47) took part in the series of focus groups facilitated in English and Spanish (25.5%). Over 40% of participants self-identified as Hispanic or Latinx, Native American, or African American with roughly 15% attending from rural areas. Similarly, professional providers age 18 to 80 (N=25) attended separate groups in either English or Spanish (48%). Our results suggest that computer-mediated focus groups offer a unique opportunity to reach diverse samples of older adults, family caregivers, and their providers. These computer-mediated focus groups also offer the chance to learn novel ways to break barriers to health access by providing virtual reach capabilities for those facing health, transportation, work, or geographic barriers.

